# Ascending aortic aneurysm and histopathology in Alport syndrome: a case report

**DOI:** 10.1186/s12882-023-03345-5

**Published:** 2023-10-12

**Authors:** Ali Kamiar, Qusai Alitter, Jose M. C. Capcha, Ali Saad, Keith A. Webster, Lina A. Shehadeh

**Affiliations:** 1 Department of Medicine, Division of Cardiology, University of Miami Leonard M. Miller School of Medicine, Miami, Fl United States; 2 Interdisciplinary Stem Cell Institute, University of Miami Leonard M. Miller School of Medicine, Miami, FL United States; 3 Departments of Pathology and Pediatrics, University of Miami Leonard M. Miller School of Medicine, Miami, FL United States; 4 Integene International Holdings, LLC, Miami, FL United States; 5 Baylor College of Medicine, Houston, TX United States; 6 Everglades BioPharma, Houston, TX United States

**Keywords:** Alport syndrome, Ascending aortic aneurysm, Kidney dysfunction, Aneurysm repair, Hypertension, Ascending aortic histopathology

## Abstract

**Background:**

Alport syndrome (AS) is caused by mutations in type IV collagen genes that typically target and compromise the integrity of basement membranes in kidney, ocular, and sensorineural cochlear tissues. Type IV and V collagens are also integral components of arterial walls, and whereas collagenopathies including AS are implicated in aortic disease, the incidence of aortic aneurysm in AS is unknown probably because of underreporting. Consequently, AS is not presently considered an independent risk factor for aortic aneurysm and more detailed case studies including histological evidence of basement membrane abnormalities are needed to determine such a possible linkage.

**Case presentation:**

Here, we present unique histopathological findings of an ascending aortic aneurysm collected at the time of surgery from an AS patient wherein hypertension was the only other known risk factor.

**Conclusions:**

The studies reveal classical histological features of aortic aneurysm, including atheroma, lymphocytic infiltration, elastin disruption, and myxoid degeneration with probable AS association.

## Background

Alport syndrome (AS) is a rare genetic disease characterized by defective production of type IV collagen, causing progressive kidney disease, sensorineural hearing loss, and ocular abnormalities [1, 2]. X-linked Alport syndrome (XLAS), the most common type of AS, which accounts for around 80 percent of diagnosed cases, is caused by mutations in the *COL4A5* gene [3]. Thoracic aortic aneurysm, a progressive pathological dilatation of the aortic wall that is life-threatening when ruptured, is typically caused by vessel wall weakness combined with hemodynamic stress and/or hypertension. Type IV collagens localize to the endothelial and smooth muscle basement membranes of the intima and media and play vital roles in vessel wall integrity [4–7]. Collagenopathies that affect type IV collagen abundance and/or function are expected to influence vascular functions [1]. Indeed, case reports have documented all categories of arterial aneurysm in AS patients, [1, 8–10] including high incidence of intracranial and intracoronary aneurysms [1]. Although hypertension is a known risk factor for aortic aneurysm, the incidence even in hypertensive subjects is rare in the absence of underlying predisposition. However, at the present time there is insufficient clinical evidence to confirm AS as an independent risk factor for aortic aneurysm or aortic dissection [3, 9, 11–14]. Similarly, the histopathology of aortic aneurysm in AS or other collagenopathies is underreported, and more studies are needed to define the molecular and genetic correlations and establish risk associations for vascular aneurysm and dissection in AS patients [1, 3]. Here we report a case study of a 63-year-old female XLAS patient who was found to have an ascending aortic aneurysm that required surgical repair.

## Case presentation

**Fig. 1:**
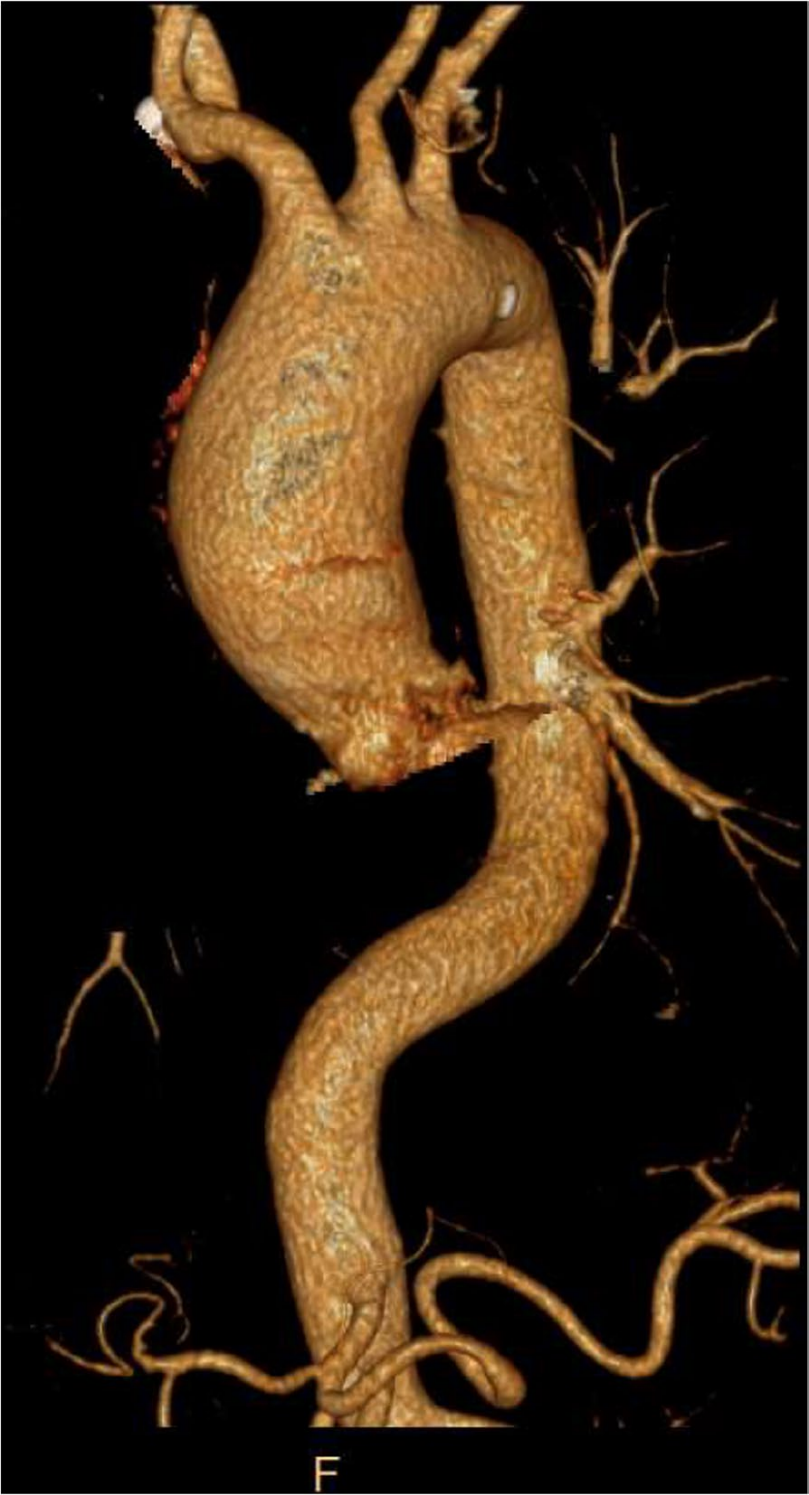
Ascending aortic aneurysm detected by imaging. Computerized tomography (CT) chest angiogram showing ascending aorta is dilated measuring maximally at 5.2 x4.6 cm cross-sectional area

A 63-year-old woman of Chinese lineage with a medical history of XLAS diagnosed at age 23, presented at the emergency room after reporting repeat chest pain and a "clammy" feeling that persisted for >2 hours. Laboratory tests showed an elevated BUN to creatinine ration of 27, (27mg/dl BUN/1.0 mg/dl creatine) consistent with possible kidney damage. The patient has a history of hypertension, and medications included lisinopril (20 mg) with hydrochlorothiazide (12.5 mg) and allopurinol (300 mg daily, for gout). The patient did not report other risk factors for cardiovascular disease. The workup, including the chest X-ray, electrocardiogram (EKG), enzyme levels, and stress tests, were normal. Outpatient cardiac calcium score imaging revealed the presence of an ascending aortal aneurysm that was confirmed by computerized tomography (CT) angiography (Fig. 1). Genetic testing of the patient at age 65 years, confirmed mutation of the XLAS-linked *Col4a5* gene (variant: c.4791T>A (p.Tyr1597*) as well as an *ABCC8* gene mutation (variant: c.4178G>A (p.Arg1393His) that can lead to metabolic disorders.

**Fig. 2:**
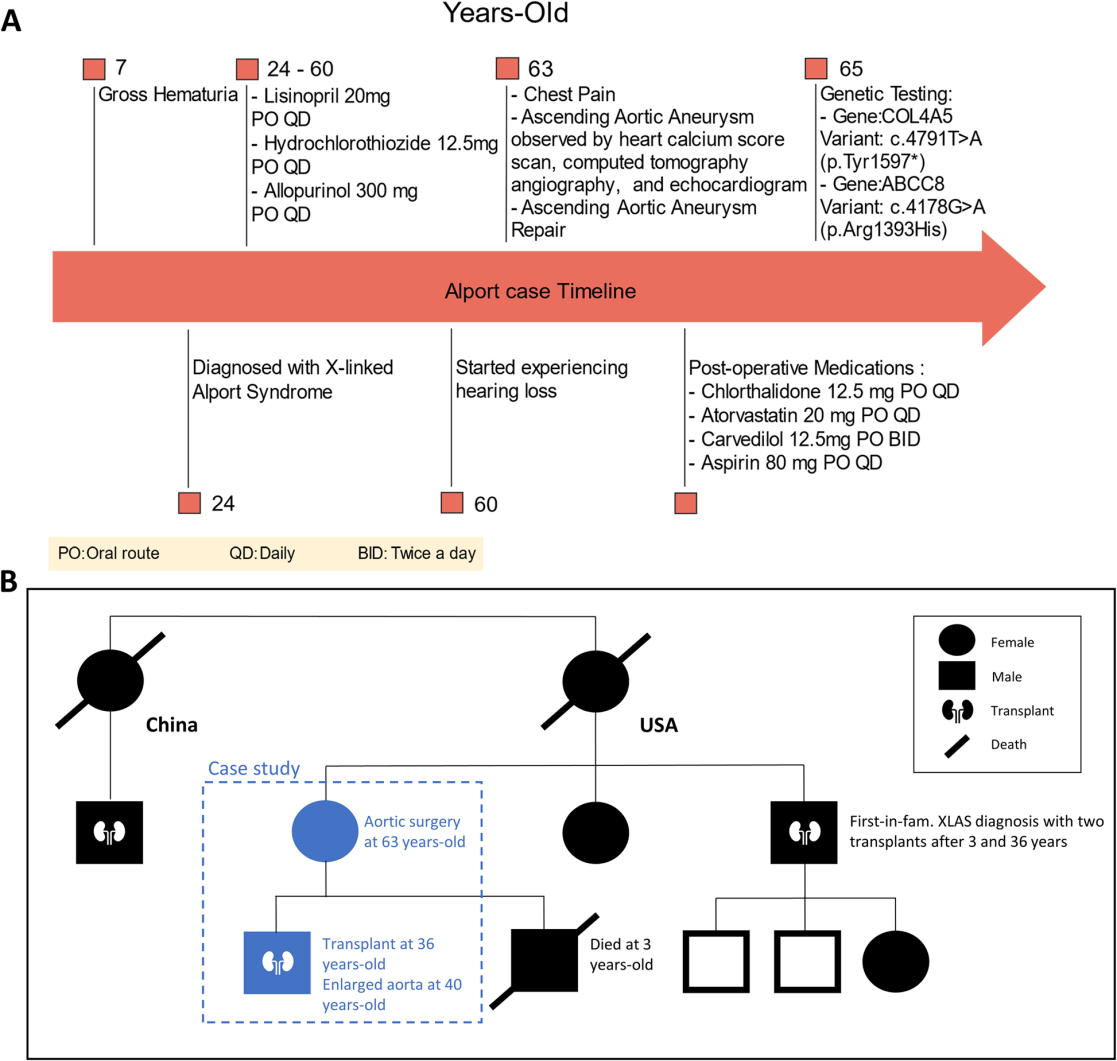
Timeline and genogram of case. Shown are the timeline of case over the years and the (**A**) and the genogram (**B**)

Echocardiography showed no evidence of significant valvular heart disease other than minimal aortic stenosis and stage I diastolic dysfunction. The patient underwent heart catheterization, which showed no evidence of significant coronary artery disease. Electively and without complications, the patient received an ascending aortic aneurysm replacement utilizing a Hemashield graft. The excised aortal tissue was collected, fixed, and stained for histopathological studies, including hematoxylin and eosin (H&E), Alcian blue, and CD45 immunostaining. After aortic aneurysm repair, her medications were changed to chlorthalidone (12.5 mg), atorvastatin (20 mg), carvedilol (12.5mg), and aspirin (80mg) per day. The case timeline is outlined in Fig. 2A.

Noteworthy, as presented in the genogram (Fig. 2B), the patient has a family history of AS, including a 55-year-old brother with severe hearing loss, chronic end-stage renal disease, and double kidney transplants. Irregularities of the brother’s aorta or presence of hypertension are not reported. The patient’s son also has an AS diagnosis, wears hearing aids, and has undergone double kidney transplantations. The son is also hypertensive and has a diagnosis of mild aortic aneurysm.

## Discussion and conclusions

There are multiple reports of aortic abnormalities in patients with AS, including aortic dilation, thoracic and abdominal aortic aneurysm, and aortic dissection [1, 3, 8–18]. The first report of aortic pathology in patients with AS described two brothers, ages 13 and 15, who were respectively diagnosed with aortic dissection and aortic root enlargement [11]. The largest case series described five patients aged 21 to 32 years old, three of whom presented with aortic dissection and aneurysm, one with asymptomatic dilation of the aorta, and one with aortic insufficiency [12]. Tayel *et al.* reported two Alport brothers with aortic abnormalities, one died of a dissecting aortic aneurysm at the age of 13 while the other suffered from both Alport and Marfanoid syndromes and was diagnosed with an asymptomatic aortic root aneurysm [11]. Lyons *et al.* reported the case of a 36-year-old male with AS, hypertension, and an active smoker who presented with bilateral flank pain due to a thoracoabdominal aortic aneurysm rupture requiring surgical intervention [9]. Other cases include a 21-year-old male with multiple asymptomatic dilatations of the ascending and descending aorta and the aortic arch, and a 32-year-old AS male with fatal rupture of the ascending aortic aneurysm [12]. It is noteworthy that the aortic abnormalities associated with AS described above occurred predominantly in young (<40 years), male subjects. The patient described here with first apparent manifestation of aortic aneurysm at age 63 years is considerably older and female. Unlike their male counterparts [19], AS females with heterozygous disease-causing COL4A5 variants are known to show a broad spectrum of clinical symptoms from mild to severe, that includes an age-dependence, with 15-30% developing end-stage kidney disease by age 60 years [20]. Skewed or preferential X-inactivation of one X chromosome has been proposed as contributing to such phenotypic variability [21–24] and could factor in the late-stage presentation of aortic aneurysm described here. Definitive proof for the contributions of such skewed X-inactivation to AS phenotype in females has not yet been achieved and is an active area of study [20]. Aortic aneurysm in the absence of AS or other underlying genetic risk factor is much more prevalent in elderly subjects reflecting the progressive and accumulative influence of other known risk factors (hypertension, atherosclerosis, inflammation) on aortic wall function [25–27].

In addition to the abdominal and thoracic aortic abnormalities associated with AS, coronary and intracranial aneurysms have also been reported. Auer *et al.* [15] reported the first case of multiple coronary aneurysms associated with AS, and suggest that defective type IV collagen is the cause. Such defective type IV collagen of AS subjects can weaken the arterial wall and reduce resistance to pulsatile forces especially with coincident hypertension. Bose *et al.* [1] recently described a high incidence of intracranial aneurysms in AS patients and suggested that hereditary collagenopathies may be linked more generally with vascular aneurysms, especially in young patients. Their results suggest that intracranial aneurysm is more prevalent in the population with collagenopathies, including AS, than previously suspected. These groups concur that epidemiological studies, additional case reports and histological analyses are warranted to define incidence and molecular genetic correlations to establish associations between AS and vascular aneurysm.

**Fig. 3:**
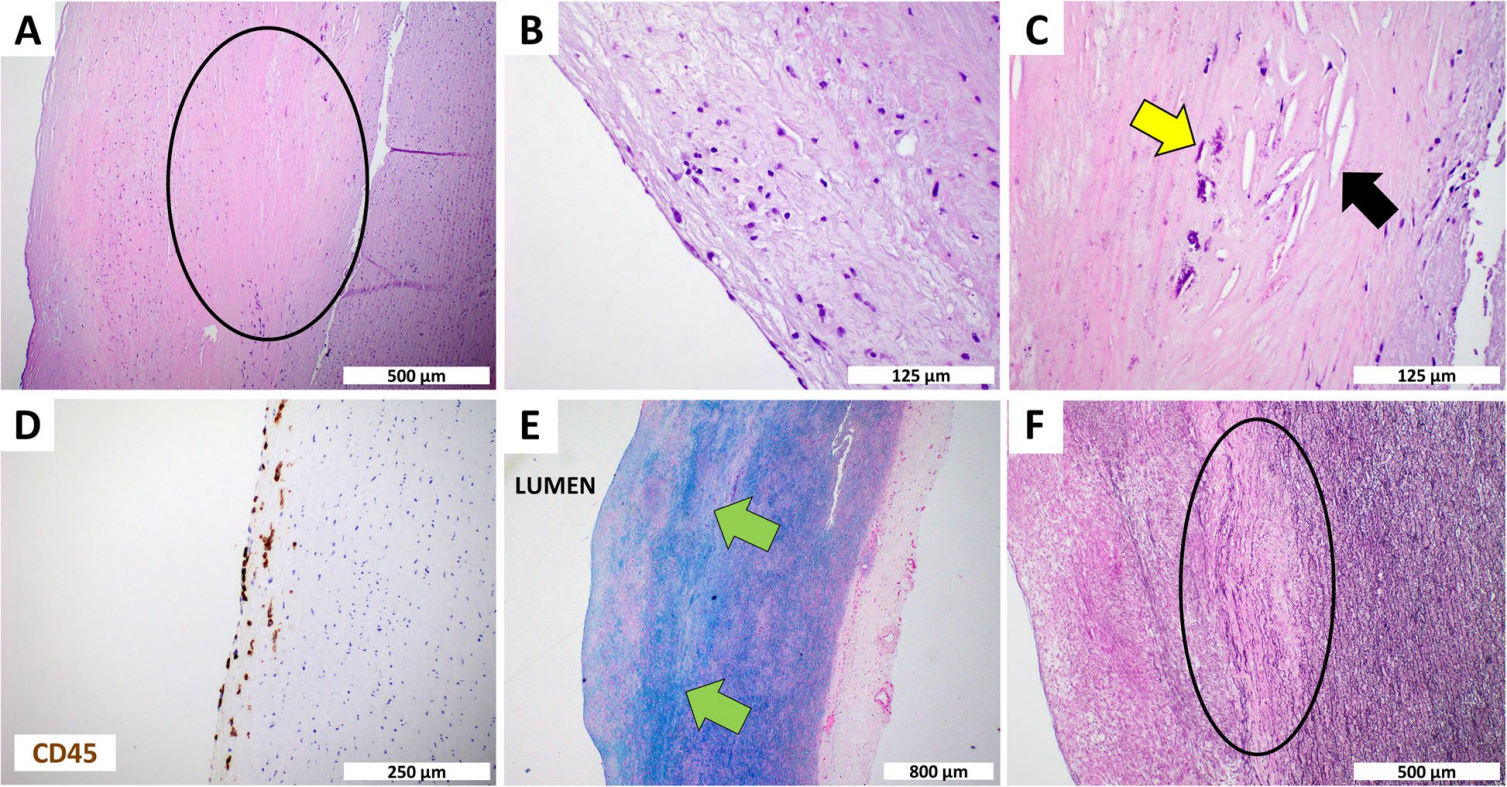
Histopathology of Aortic Dissection in Alport Patient. H&E shows (**A**) Medial Necrosis (circle), (**B**) Scattered lymphocytes in the intima, and (**C**) Atherosclerotic plaque composed of calcifications (yellow arrow) and cholesterol clefts (black arrow). **D** CD45 shows intimal lymphocytic infiltration, (**E**) Alcian blue shows marked myxoid degeneration (green arrow) in the intima, and (**F**) Elastin shows disruption of elastic fibers (circle)

A CT angiography of our patient showed that the ascending aorta is dilated with a maximal cross-sectional area of 5.2 x 4.6 cm. H&E staining (Fig. 3) demonstrates medial necrosis, dispersed infiltration of lymphocytes, and atheroma. Anti-CD45 staining confirmed intimal leukocyte infiltration. Inflammation is a hallmark of aneurysm progression, with samples from aortic aneurysm patients showing progressive inflammatory cell infiltration of both innate and adaptive immune cells [19]. Atheroma is characterized by calcification, accumulation of cholesterol and lymphocytes and exacerbated extracellular matrix synthesis [28]. Although atheroma is evident in our patient samples (Fig. 3C), and has been linked with aortic aneurysm, it is not clear whether the atheroma has an active role in aorta dilation or disease progression [29]. Destructive changes in the aortic wall connective tissue can also cause aortic enlargement during the early stages of aneurysmal development. Imbalanced connective tissue repair and degradation play an important role in aneurysm growth [30]. Aside from atherosclerosis, myxoid degeneration may cause aneurysms with possible rupture [31]. Myxoid degeneration is a degenerative process in which the connective tissues are replaced by primitive myxoid connective tissue [31]. As shown in Fig. 3, Alcian blue stain shows marked myxoid degeneration. In addition, our elastin staining shows disruption of elastic fibers. Elastin is one of the most abundant extracellular proteins in the aorta and has an important role in wall elasticity and flexibility [30]. In a recent case study of chronic type A aortic dissection in a 39 year old hypertensive AS woman, Nishiori *et al.* [3], reported the presence of fragmented and disorganized collagen alpha 5 chains, severely disorganized elastic fibers and mild mucinous changes in the tunica media of the aortic wall, strongly implicating AS in the etiology of this case of aortic dissection.

To our knowledge, this is the first case study to report histopathological findings of an AS patient who survived preemptive surgery of an ascending aortic aneurysm. The results confirm classic features of inflammatory cell infiltration, medial necrosis, disruption of elastin fibers, and myxoid degeneration associated with AS-related ascending aortic aneurysm. Although other genetic disorders such as Marfan, Ehlers Danlos, Turner, and Loeys-Dietz syndrome are listed as risk factors for aortic aneurysms and dissections, AS has not yet achieved such a high-risk status for aortic disease. Our case report supports the growing evidence for such a linkage between AS and aortic disease and highlights the importance of angiographic and abdominal ultrasound screening for aortic aneurysm in patients with AS, even with controlled blood pressure and a healthy lifestyle. Mutation of ABCC8, a gene that encodes a subunit of the ATP-sensitive potassium channel has been associated with various forms of diabetes mellitus and hyperinsulinemia that can affect kidney related function and disease [32–34]. Roles for ABCC8 mutation in Alport related aneurysm have not been reported.

## Data Availability

All the data and materials are available. Lina A Shehadeh should be contacted if someone wants to review the data from this study.

## Abbreviations

AS: Alport syndrome
XLAS: X-linked Alport syndrome
EKG: Electrocardiogram
CT: Computerized tomography
H&E: Hematoxylin and Eosin staining
